# The views of healthcare professionals and family members on deprescribing preventive medication in people living with dementia: a qualitative systematic review

**DOI:** 10.1186/s12877-026-07480-0

**Published:** 2026-04-18

**Authors:** Clare Bates, Nikolaos Efstathiou, Claire Sutton, Nesrein Hamed, Ian Maidment

**Affiliations:** 1 Pharmacy School, Aston University, Birmingham, England; 2 School of Nursing and Midwifery, University of Birmingham, Birmingham, England

**Keywords:** Deprescribing, Preventive medication, Dementia, Qualitative systematic review, Advance care planning, Decision-making, Mental capacity, Life-sustaining, Life-prolonging, Advance decision to refuse treatment

## Abstract

**Background:**

People living with dementia often continue to take preventive medication until the end of their lives, largely with the aim of prolonging life. However, at the later stages of dementia, a palliative-only, symptom-controlling approach, may be more appropriate. Also, the efficacy of preventive medications in older people is often unclear and the side effects may have a negative impact on quality of life, yet these medicines are often not deprescribed.

**Aim:**

To synthesise qualitative evidence of healthcare professionals’ and family members’ views on deprescribing preventive medication for people living with dementia.

**Methods:**

The systematic review was conducted using the principles for systematic reviews of qualitative evidence provided by Cochrane. In May 2025, keywords were searched for in Embase, Health Management Information Consortium, MEDLINE, PsycINFO, Cumulative Index to Nursing and Allied Health Literature, PubMed, Open Access Theses and Dissertations, Web of Science, ProQuest, Cochrane Central Library and Google Scholar to identify suitable studies from pre-defined eligibility criteria. The Critical Appraisal Skills Programme was used for quality appraisal and Thomas and Harden’s qualitative thematic synthesis for synthesising findings.

**Results:**

Eighteen studies were included. Five themes were identified: the decision-makers, reactive reasons to deprescribe, barriers and facilitators to proactive deprescribing, difficulties for family members and the timing of deprescribing decisions. Guidance is often unclear for this complex group, but guidance is unlikely to cover every individuals’ unique situation. Ideally, in practice, deprescribing should occur before someone gets to a stage when they can no longer communicate side effects and this may instigate more deprescribing discussions. To assist deprescribing, recording why a medication was started on the prescription could make it clearer whether to discontinue it at a later stage.

**Conclusion:**

Deprescribing conversations are difficult for both healthcare professionals and family members. Future research could explore the role of advance care planning for deprescribing preventive medications, before someone is diagnosed with dementia, so decision-makers are aware of what the person would have wanted. This may make deprescribing less time-consuming, provide guidance and reduce the burden for the decision-makers.

**Trial registration:**

PROSPERO CRD42023476394 (October 2023).

**Supplementary Information:**

The online version contains supplementary material available at 10.1186/s12877-026-07480-0.

## Introduction

In the United Kingdom (UK), 55% of people are expected to be directly affected by dementia, either by having the illness themselves or by being the main carer for a family member (or both) [1]. There are currently nearly one million people/person living with dementia (PLWD) in the UK [2] and more than half of these people have severe dementia [3]. Worldwide, in 2020, there were over 50 million PLWD and this is expected to increase to 152 million by 2050 [4].

Dementia is a degenerative, long-term illness, and most people diagnosed with dementia will survive for 8–10 years [5]. At diagnosis, 82% of people will be taking three or more medications for their other illnesses [6] and in their last year of life most people will still be taking preventive medication [7]. Preventive medication (as opposed to medication for symptom-relief) are largely intended to prolong life, examples of which include statins, antihyperglycemics, antiplatelets and antihypertensives [8]. PLWD may lack decision-making capacity on whether they would like to continue preventive medication or commence a palliative-only approach to treatment. They may also be unable to make decisions on whether the benefits of medication outweigh the side effects.

Older people are at a greater risk of medication-related adverse events, including side effects. They tend to take more medications than younger people, causing an increase in the risk of drug-drug and drug-disease interactions [9]. Physiological changes often occur as people age, including reduced liver and kidney function, affecting the pharmacokinetics and pharmacodynamics of medication [10]. Clinical trials rarely include older people, so the efficacy and safety of medications is often unknown in this age group [11]. In 2022, a UK study showed 16.5% of hospital admissions were due to adverse drug reactions with higher rates in older people and those with co-morbidities including dementia [12]. There is also an increased risk of medication errors in PLWD, due to their cognitive impairment [13]. However, deprescribing is often not considered [7].

Deprescribing is defined as reducing or withdrawing medications that are considered inappropriate [14]. Deprescribing discussions are complicated because of a lack of evidence on the risks and benefits of taking medication in older people and is further complicated by PLWD’s mental capacity. Lui et al. [15] showed that medication decision-making was often impaired before PLWD were even diagnosed with dementia. Other stakeholders (general practitioners [GPs], nurses, pharmacists, and family members) then have to make these complex deprescribing decisions [16].

This is the first systematic review to focus on stakeholders’ views on deprescribing preventive medication in dementia. Previous qualitative systematic reviews about deprescribing in end-of-life situations have not focused on dementia specifically [17–19]. These systematic reviews found that deprescribing was the primary responsibility of the GP and was hindered by a lack of time within their busy workloads. The GPs’ confidence in their abilities, with the worry of unwanted consequences for themselves or the patient, coupled with pressure from patients, relatives and other health care professionals, made deprescribing decisions difficult. Furthermore, they reported that guidelines for deprescribing were too complex or unclear, and disease-specific guidelines encouraged prescribing.

Other systematic reviews that incorporate deprescribing and dementia use quantitative evidence, looking at outcomes of deprescribing and the effects of various interventions [20, 21]. For this review qualitative evidence was required, as views and experiences are subjective and explore meanings and context, and therefore cannot be captured in-depth by numerical and quantitative evidence. The aim of this review was to identify, appraise and synthesise qualitative evidence relevant to the research question: What are the key stakeholders’ views and experiences on deprescribing preventive medication in PLWD?

The objectives of the systematic review were to:

1. Identify differences in stakeholders’ views on deprescribing preventive medication including the stage of dementia and setting.
2. Find who is best placed to make deprescribing decisions in this population.
3. Make recommendations for education, policy, practice and research with a focus on how best to incorporate the beliefs and attitudes of the PLWD into deprescribing decision-making.

## Methods

The Cochrane principles for systematic reviews of qualitative evidence were followed. This included Patient and Public Involvement developing the research question and contributing to the discussion [22]. The ENTREQ (Enhancing transparency in reporting the synthesis of qualitative research) statement [23] (Appendix 1) was followed and the PRISMA (Preferred Reporting Items for Systematic reviews and Meta-Analyses) flow diagram and checklist (Appendix 4) was used [24]. The protocol has been published in BMJ Open [25] and was registered on PROSPERO (CRD42023476394).

### Search strategy

The review team with the support of an experienced medical librarian selected the search terms and databases. The following electronic bibliographic databases were searched: Embase, Health Management Information Consortium, MEDLINE, PsycINFO (Ovid platform); Cumulative Index to Nursing and Allied Health Literature, PubMed (EBSCO platform); ProQuest Central and Proquest; Cochrane Central Library; Web of Science (for citation mining of final selected papers) and Open Access Theses and Dissertations. Appendix 2 is an example of the search strategy used for Embase.

The search was conducted using synonyms of the following words and the Boolean operators “AND” and “OR”:

- dementia OR elderly OR mental capacity OR nursing home.

AND

- deprescribing OR withdrawing medication.

AND

- personal experience OR view OR qualitative.

AND

- healthcare professional OR family member.

Truncation and MeSH terms were also used.

The reference lists of included papers and any similar systematic reviews were searched for relevant studies. Deprescribing networks, dementia organisations’ websites and relevant conference material were also checked. The first 200 hits on Google Scholar were screened and continued until 10 consecutive irrelevant results were found. Grey literature was also searched for in OpenGrey and the National Grey Literature Collection.

Pre-defined inclusion and exclusion criteria were used (Appendix 3). The inclusion criteria were studies that incorporated any people involved in the decision-making process of deprescribing for PLWD and their views of this subject. The exclusion criteria were any studies that did not clearly state that findings were specifically related to PLWD or were not referring to deprescribing preventive medication.

Due to the low number of articles that were specifically about PLWD and deprescribing preventive medication, it was decided amongst the review team that articles that were for a more general group of older people would be included in the search. However, only the segments that could be identified in the findings as being related to PLWD would be coded. Likewise, studies that also included medication used for symptom-control, such as opioids, would only have the segments related to preventive medication coded. In studies that related to wider medication management for PLWD, rather than just deprescribing, only segments that related to deprescribing would be included in the coding.

There was no date restriction as there was minimal research involving deprescribing before 2014 [26]. The search was started and completed in May 2025. Only English language studies were included due to lack of funding for professional translators.

Primary qualitative studies and studies reporting on secondary analysis of qualitative data were included. Quantitative studies, audit/service evaluations, systematic reviews, protocols, books, editorials, and opinion pieces were excluded.

### Selection process

Most of the duplicate citations were identified and removed by Covidence (a web-based systematic review management system). Any remaining duplications were removed manually by the reviewers. Using Covidence, two reviewers (CB, NH) independently screened titles and abstracts and removed any articles that did not meet the eligibility criteria. The same two reviewers then independently read all the remaining articles in full text (including articles found through any other means, for example, citation mining) to decide whether they met the eligibility criteria. An independent third reviewer (IM) acted as arbitrator.

### Quality assessment

The CASP (Critical Appraisal Skills Programme) [27] Qualitative Studies Checklist was used to appraise the qualitative studies. CASP was chosen because it is a well-recognised tool designed for health research and is most frequently used in systematic reviews for qualitative studies [28]. The first reviewer (CB) appraised all studies, and a second reviewer (NH) appraised 30% independently. When consensus was reached on the use of the CASP tool, the first reviewer continued to appraise all other included studies. A third reviewer (IM) arbitrated as required.

### Strategy for synthesis

An inductive approach was used to allow new ideas to be generated, and Thomas and Harden’s [29] thematic synthesis was used which is suitable for all types of qualitative and inductive research [30]. NVivo software was used to assist with coding the data, organising themes and keeping an audit trail [31].

Coding was performed by the first reviewer, who “line-by-line” coded the first two included studies’ findings. Each piece of data extract was given one or more codes, creating a new code if one did not already exist. This was repeated independently by the second reviewer and any disagreements resolved through discussion, however there were no significant differences between the two reviewers’ codes. The two reviewers then discussed their initial coding with the third reviewer to ensure a robust approach was used. The first reviewer then coded the findings from the other studies, which were then reviewed by the whole team. Codes were then organised into themes and sub-themes. The first reviewer met with the review team fortnightly to discuss the synthesis development and any issues that arose, including those recorded in the reviewer’s reflexivity journal; this was to reduce bias and error [32].

## Results

**Fig. 1:**
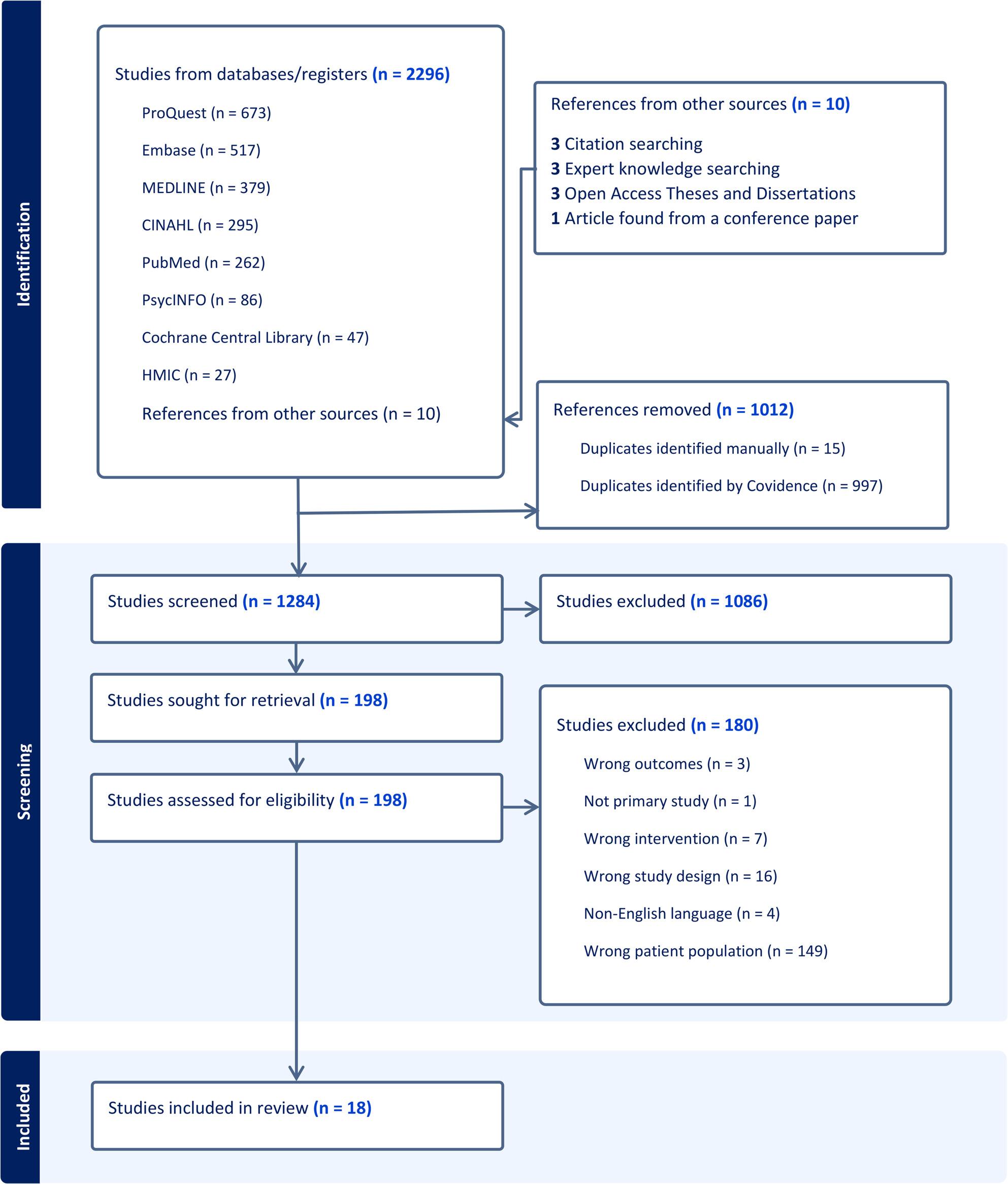
PRISMA flow diagram

Figure 1 shows the screening process using the PRISMA flow diagram [24]. 2296 studies were retrieved, and 18 were included in the final selection. Of these, eleven studies were specifically about PLWD, and seven studies had segments that were identified as relating to PLWD and only those segments were coded.

### Study characteristics

The studies were conducted in Australia (*n* = 3), Denmark (*n* = 1), Netherlands (*n* = 1), Sweden (*n* = 1), UK (*n* = 3), USA (United States of America) (*n* = 8) and one study involved a global online forum (Table 1). Nurses (*n* = 2), psychologists (*n* = 2), pharmacists (*n* = 8), and doctors (*n* = 6) led the 18 studies in this systematic review.

The interviewees were doctors, nurses, pharmacists, family members and PLWD. Most studies used semi-structured interviews, although focus groups were also used, and two were qualitative content analyses of GP or pharmacist consultations. The studies were based in care homes, hospitals, hospices, community, outpatients and academia. Most studies focused on general deprescribing, but four focused on deprescribing specific medications: bisphosphonates (three studies) and anti-dementia medications (one study). Most of the data were collected between 2014 and 2023. However, the online forum had data going back to 2005.

**Table 1:** Summary of studies and reported study results

Author/s, country and setting	Aims and objectives of the study	Population interviewed/surveyed (which healthcare professional or type of family member)	Methods (including sampling strategy, data collection and analysis)	Findings/themes from the study
**STUDIES RELATING SPECIFICALLY TO PLWD**				
Antonelli et al. (2022) [33] USA Community-dwelling people	To explore whether mailing educational materials to people with Alzheimer’s disease and related dementias might encourage conversations with their healthcare provider about potentially inappropriate medications.	27 interviews: 9 with caregivers only, 2 with patients only, 3 with patient-caregiver dyads, and 13 with GPs	Semi-structured interviews Convenience sample Inductive thematic analysis	1. if a medication might cause harm, it would motivate conversations about deprescribing 2. trust in the provider could facilitate or inhibit such conversations; conversations would be more likely if there were prior positive experiences 3. providers were receptive to patients and caregivers initiating conversations about their medications
Cross et al. (2021) [34] Australia Community and GP settings	To explore stakeholder roles in medication management for people with dementia, including barriers and enablers to achieving those roles.	14 consumers (2 people with dementia and 12 carers of people with dementia), 22 general practitioners (GPs), 9 registered nurses and 10 pharmacists	*Secondary data analysis* Focus groups Thematically analysed using an inductive approach	1. supporting the role of the person with dementia 2. carer roles and challenges 3. health professional roles 4. process and structure barriers to medication management
Disalvo et al. (2020) [35] Australia Long-term care facilities	To explore medication-related decision-making by health professionals from different disciplines and specialties caring for people with advanced dementia living in long-term care facilities, focusing on starting, continuing or deprescribing medications commonly regarded as potentially inappropriate.	8 Physicians, 5 Nurses, 3 Pharmacists	Focus groups Qualitative analysis using line-by-line inductive coding Person-centred framework	1. regardless of medication type or dilemma, results showed that decision-making for people with advanced dementia should start with discussing goals of care 2. families need to be involved 3. it is an iterative process that requires regular monitoring and adjustment
Fasth et al. (2025) [36] North Carolina, USA Nursing homes, community or long-term care	To characterize and compare communication preferences of prescribers and family/informal caregivers regarding deprescribing of bisphosphonates.	10 physicians and 2 nurse practitioners plus 11 family or informal caregivers of older adults living with dementia	*Secondary data analysis of* **Niznik** et al. **(2023a) and Niznik** et al. **(2023b)** Semi-structured interviews Qualitative framework analysis	Deprescribing discussions were preferred if they focused on: 1. risk versus benefit in relation to patient-specific factors, such as functional status, goals of care and indication for treatment. 2. caregivers wanted information of the impact on cognition. The least preferred conversation starter for deprescribing was: 1. the extra effort and cost of continuing bisphosphonates. 2. caregivers disliked statements that introduced discussions of prognosis and life expectancy. For deprescribing to be framed as a form of medication optimization and not a withdrawal of care.
Green et al. (2019) [37] USA Primary care and outpatient clinics	To investigate clinician-perceived barriers and facilitators of reducing polypharmacy and potentially inappropriate medicine use in people with dementia.	21 clinicians from 16 different clinics participated (19 physicians and 2 nurse practitioners)	Purposive, or expert, sampling and snowball sampling Semi-structured interviews Transcripts were analysed using qualitative content analysis	Barriers to deprescribing: 1. certain medications are more complicated to deprescribe: oral anticoagulants, antidiabetic agents, statins, bladder antimuscarinics, and antipsychotics 2. lack of evidence on the efficacy and safety of most medications in people with dementia 3. difficulty assessing medication effects in people with dementia 4. the perception that deprescribing medication is seen as “giving up” on someone Facilitators to deprescribing: 1. Access to interdisciplinary services 2. Guidelines for nondementia illnesses (e.g. diabetes) addressing the care of people with dementia
Green et al. (2020a) [38] USA Integrated delivery system (includes hospitals, clinics and physician practices) in Colorado and an academic medical centre in Maryland	To elicit stakeholder perspectives on the content and process of deprescribing communication in primary care, focusing on African American and Hispanic patients.	Interviews with 17 patients, 16 caregivers, and 16 physicians	Convenience, purposive and snowball sampling Semi-structured interviews Inductive coding and deductive coding were used for the analysis	1. earn trust before deprescribing 2. frame deprescribing as routine and positive 3. align deprescribing with the goals of dementia care 4. respect caregivers’ expertise 5. racial, ethnic, and language concordance was important to patients and caregivers from minority cultural backgrounds, and physicians wanted communication tips addressing specific clinical situations 6. follow-up during the deprescribing process 7. deprescribing explained in terms of altered physiology in ageing was preferred by patients and caregivers
Green et al. (2025) [39] USA Integrated delivery system (includes hospitals, clinics and physician practices) in Colorado and an academic medical centre in Maryland	To gain in- depth knowledge of how elicitation of PLWD and care partner medication- related priorities during a deprescribing intervention shaped discussions with pharmacists about medications.	82 transcripts from encounters with 55 patient-carer dyads, seen by one of two geriatric-trained pharmacists	*Secondary data analysis* Qualitative content analysis of audio- recorded interactions from a pilot study	1. Reducing medication- related treatment burden 2. Alleviating burdensome symptoms 3. Maintaining cognition and function 4. Discussion of trade-offs 5. Challenges to deprescribing
McCloskey (2016) [40] Northern Ireland Primary care, secondary care, hospice care, academia	To explore decision-making, approaches to care, prescribing and expectations in relation to medication use for people with advanced dementia approaching the end of life, from the perspectives of healthcare professionals and those involved in making decisions for the person with advanced dementia (carers, family members and relevant others).	8 General Practitioners, 12 Hospital Geriatricians, 1 Hospice Doctors, 12 Pharmacists and 15 Key Decision-Makers for PLWD	Semi-structured Interviews Thematic analysis	Physicians were driven by ‘getting the balance right’; this involved: 1. patient- and drug-specific factors 2. place of care 3. communication with other healthcare professionals 4. involvement of family (who expect to be informed in medication decisions of their loved one and that comfort is a priority as dementia progresses) Palliative care pharmacists were comfortable delivering palliative care to people with advanced dementia approaching the end of life, and community pharmacists expressed a desire to become more involved.
Niznik et al. (2023a) [41] North Carolina, USA Nursing homes	To identify factors influencing prescriber decision-making for deprescribing bisphosphonates for older nursing home residents with dementia.	10 physicians and 2 nurse practitioners	Snowball referral approach Semi-structured interviews Qualitative analysis framework methodology and the social-ecological framework guided coding	Reasons to deprescribe: 1. prior bisphosphonate treatment course of several years 2. emergence of adverse effects 3. changing goals of care or limited life expectancy Reasons deprescribing does not happen: 1. family perceive deprescribing as “withdrawing care” 2. frequent transition between care providers makes it difficult to obtain the correct medication history 3. lack of guidelines (or deprescribing criteria) specific to dementia or immobile residents
Niznik et al. (2023b) [42] North Carolina, USA Community or long-term care	To better understand the decision-making processes of caregivers of older adults living with dementia for continuing versus deprescribing bisphosphonates.	11 family or informal caregivers of older adults living with dementia	Semi-structured interviews Qualitative analysis framework methodology guided by the Health Belief Model	Reasons for taking bisphosphonates: 1. to maintain functional independence (although this may become less important near the end of life) 2. improved quality of life Reasons to deprescribe: 1. gastrointestinal adverse effects 2. preference for fewer treatments 3. dementia-related symptoms that affect medication administration
Parsons and Gamble [43] (2019) Worldwide Web 2005–2017	To investigate the experiences and perspectives of carers and family members when antidementia medications (cholinesterase inhibitors and/or memantine) are stopped.	Generally, carers and family members of people with dementia: 95 threads comprising posts from 112 users	Archived discussions from Talking Point (an online discussion forum hosted by the Alzheimer’s Society UK) Thematic analysis using the Framework method	1. expectations about withdrawal 2. method of withdrawal 3. clinical condition on withdrawal 4. the effect of withdrawal on caregivers
STUDIES THAT ONLY SECTIONS OF THE FINDINGS RELATE TO PLWD				
Birt et al. (2022) [44] UK Care homes	To explore primary care pharmacists, GPs and care home managers’ beliefs and practices of proactive deprescribing in care homes.	pharmacist-Independent prescribers (PIPs, *n* = 16), General Practitioners (GP, *n* = 6) and care home managers (CHM, *n* = 7)	Semi-structured interviews Purposive selection framework Theoretical Domains Framework Reflexive thematic analysis	1. multidisciplinary team and routine practices in GP surgeries and care homes affected deprescribing 2. an understanding of the resident’s medical history made it easier to balance the risks of deprescribing
Bolmsjö et al. (2015) [45] Sweden Nursing homes	To explore the GPs’ work with the elderly in nursing homes to provide input on how the care can be improved and identify potential obstacles for good quality of care.	12 GPs interviewed, focus group discussion with 6 of the interviewed GPs	Semi-structured interviews Follow-up focus group discussion Systematic text condensation analysis	1. concern for the patient (the continuum of ageing, care needs and medicalisation, view of the nurse as a key person for the patient’s well-being) 2. sustainable working conditions (holism, collaboration, freedom and variation, and meaningfulness)
Green et al. (2020b) [46] Baltimore, Maryland. USA 2 primary care clinics and 1 hospital-based geriatric clinic	To characterise how primary care clinicians discuss medications during encounters with older adults with cognitive impairment and their companions.	93 patient–companion dyads seen by 14 different clinicians (physicians, nurse practitioners, or physician assistants)	*Secondary data analysis* Qualitative content analysis Constant comparative approach Open coding allowed inductive and deductive coding Shared decision-making frameworks	1. there are numerous ways in which primary care clinicians introduce patients and companions to key principles of optimal prescribing and deprescribing 2. clinicians used a variety of approaches to foster shared decision-making about medication use. 3. several challenges prevented clinicians from working with patients and companions to optimise prescribing and deprescribing
Lundby et al. (2020) [47] Southern Denmark Primary and secondary care settings	To explore different Health Care Practitioners’ perspectives on deprescribing in older patients with limited life expectancy.	6 focus groups, each with 4–6 participants (32 altogether). Group a) Family Physicians, (b) geriatricians, (c) clinical pharmacologists, (d) clinical pharmacists, (e) nurses and (f) healthcare assistants	Purposive sampling Focus groups Semi-structured interviews Systematic text condensation Phenomenological-hermeneutical approach	1. approaching deprescribing 2. taking responsibility 3. collaboration across professions
Reeve et al. (2016) [48] New South Wales, Australia Community and residential care settings	To explore the views, beliefs, and attitudes of older adults and carers on deprescribing.	8 non-paid carers in the community (focus group 1), 6 non-paid carers in a residential care setting (focus group 4), 11 older adults living in the community (focus group 2), 3 older adults from a retirement village (focus group 3)	Four focus groups Purposive sampling Directed content analysis with additional conventional content analysis	1. their perception of the appropriateness of that medication 2. fear of outcomes of withdrawal 3. dislike taking medications 4. the availability of a process for withdrawal (knowing that the medication could be restarted if necessary) A unique theme to the carers was the complexity of deprescribing
Warmoth et al. (2023) [49] UK 15 care homes from 2 organisations	To explore the factors that may help or hinder deprescribing practice for older people within care homes.	23 care home staff, 8 residents, 4 family members and 1 general practitioner	Purposive sampling Semi-structured interviews Framework analysis informed by the Consolidated Framework for Implementation Research	1. deprescribing is a complex process 2. internal and external contextual factors influencing deprescribing practice (such as beliefs, abilities and relationships) The quality of local relationships with and between healthcare professionals was more important than the type of care home management structure.
Wouters et al. (2019) [50] Netherlands Nursing homes	To examine the barriers and facilitators of conducting medication reviews aimed at improving medication prescribing in nursing home residents.	6 nursing home residents/relatives of nursing home residents, 8 elder care physicians, 5 pharmacists, and 10 nurses took part in the semi-structured interviews	Purposive sampling Semi-structured interviews Analysed with the “method of constant comparison”	1. “realising fidelity of the patient perspective…(a delicate balance between the value and the impediments of a proper assessment of the patient perspective)” 2. “level of comprehensiveness of medication reviews” 3. “inclinations of healthcare providers” 4. “inter-professional collaboration and alliances”

### Quality appraisal

All 18 studies had at least seven out of the ten criteria of the CASP checklist (Table 2) marked as “Yes”, meaning they were of a satisfactory standard for those criteria. Several studies’ recruitment (question 4) was classed as “Unsure” as they had interviewed people they knew personally or who had been involved in a previous deprescribing study that may have created bias. Others had interviewed small numbers of GPs, pharmacists, or nurses, which the review team did not consider adequate numbers to represent their professions. The relationship between the researcher and the participants (question 6) was often poorly explained or not reported at all.

**Table 2:** Quality appraisal of studies using the CASP checklist

	1. Was there a clear statement of the aims of the research?	2. Is a qualitative methodology appropriate?	3. Was the research design appropriate to address the aims of the research?	4. Was the recruitment strategy appropriate to the aims of the research?	5. Was the data collected in a way that addressed the research issue?	6. Has the relationship between researcher and participants been adequately considered?	7. Have ethical issues been taken into consideration?	8. Was the data analysis sufficiently rigorous?	9. Is there a clear statement of findings?	10. How valuable is the research?
Antonelli et al. (2022) [33]	✓	✓	✓	✓	✓	−	✓	✓	✓	✓
Birt et al. (2022) [44]	✓	✓	✓	−	✓	✓	✓	✓	−	✓
Bolmsjö et al. (2015) [45]	✓	✓	✓	−	✓	✓	✓	✓	✓	✓
Cross et al. (2021) [34]	✓	✓	−	✓	✓	−	✓	✓	✓	✓
Disalvo et al. (2020) [35]	✓	✓	✓	−	✓	✓	✓	✓	✓	✓
Fasth et al. (2025) [36]	✓	✓	−	✓	✓	−	✓	✓	✓	✓
Green et al. (2019) [37]	✓	✓	✓	✓	✓	−	✓	✓	✓	✓
Green et al. (2020a) [38]	✓	✓	✓	✓	✓	−	✓	✓	✓	✓
Green et al. (2020b) [46]	✓	✓	−	−	✓	✓	−	✓	✓	✓
Green et al. (2025) [39]	✓	✓	✓	✓	✓	−	−	✓	✓	✓
Lundby et al. (2020) [47]	✓	✓	✓	✓	✓	✓	✓	✓	✓	✓
McCloskey (2016) [40]	✓	✓	✓	✓	✓	−	✓	✓	✓	✓
Niznik et al. (2023a) [41]	✓	✓	✓	✓	✓	✓	✓	✓	−	✓
Niznik et al. (2023b) [42]	✓	✓	✓	✓	✓	✓	✓	✓	✓	✓
Parsons and Gamble (2019) [43]	✓	✓	✓	−	−	✓	✓	✓	✓	✓
Reeve et al. (2016) [48]	✓	✓	✓	−	−	✓	✓	✓	✓	✓
Warmoth et al. (2023) [49]	✓	✓	✓	−	✓	−	✓	−	✓	✓
Wouters et al. (2019) [50]	✓	✓	✓	✓	✓	✓	✓	✓	✓	✓

✓ = Yes

− = Unclear

As all studies were ethically approved and peer-reviewed, and as no study in the CASP assessment was significantly poorer than the others, all studies were given equal weighting in the synthesis. Due to the large volume of quotes documented in the 18 studies, it was decided by the review team that the findings from the review would stem from direct quotes (participants’ views) rather than their interpretations (authors’ views). This would increase the authenticity of the review.

### Review findings

#### The decision-makers involved in deprescribing for PLWD

In thirteen studies (Table 3), the GP was the overarching person responsible for deprescribing decisions. Different people (e.g. a nurse in a care home, a worried family member, or a pharmacist concerned about drug interactions) might initiate a deprescribing discussion, but the GP is accountable for the overall decision. GPs have a general understanding of the various body systems, diseases and treatments. They combine all the medications from the different specialists involved with the PLWD (checking for drug-drug and drug-disease interactions) and issue repeat prescriptions. They understand the patients in the context of their environment and may also have known them before they developed dementia and can, therefore bring this knowledge together to support deprescribing decisions (34). However, some countries, have initiatives to encourage nurse and pharmacist independent prescribers, who may take on more of this responsibility (35).

**Table 3:** Themes, sub-themes and which studies incorporate these

Theme	Sub-theme	Antonelli et al. (2022) [33]	Birt et al. (2022) [44]	Bolmsjö et al. (2015) [45]	Cross et al. (2021) [34]	Disalvo et al. (2020) [35]	Fasth et al. (2025) [36]	Green et al. (2019) [37]	Green et al. (2020a) [38]	Green et al. (2020b) [46]	Green et al. (2025) [39]	Lundby et al. (2020) [47]	McCloskey (2016) [40]	Niznik et al. (2023a) [41]	Niznik et al. (2023b) [42]	Parsons and Gamble (2019) [43]	Reeve et al. (2016) [48]	Warmoth et al. (2023) [49]	Wouters et al. (2019) [50]
The Decision-Makers Involved in Deprescribing for PLWD	GP as overall decision-maker	✓			✓ *	✓	✓	✓	✓	✓	✓		✓	✓	✓	✓			✓
	“The GPs, we’re central. So we’re the common denominator between all the different specialties, because each specialist is looking after a particular organ system and doesn’t necessarily understand the other ones, doesn’t necessarily understand the patient’s community setting and family, and hasn’t known them premorbidly like we do. So we’re the one who really needs to make sure that all these medications are working together and are needed and are doing more good than harm. We’re the ones who communicate with the pharmacist much more than the specialist.” (GP 8)																		
	Family member as advocate/ expert of PLWD	✓			✓		✓						✓ *	✓	✓	✓	✓		✓
	“Your prime responsibility is the individual patient but you can prescribe best when you have the most information that you can possibly have. So taking on board what families say and what they understand their loved one would want.” (Hospice Doctor 13)																		
	Nurse as advocate/ expert of PLWD/ as clinical expert		✓		✓	✓	✓						✓					✓	✓ *
	“Well often initiation of medications is discussed [without the nurse being present]. But then, you do not know precisely if a patient has constipation, or difficulties with taking their medications. The physician does not always know this … The nursing staff does.” (Pelican, Pharmacist, care home, discussing case 1439)																		
	Pharmacist as expert				✓	✓			✓				✓ *	✓					
	“I suppose when we talk about advanced dementia and end of life our role as community pharmacists could be described as a go-between…a go-between for doctors, nurses and patient’s families.” (Community Pharmacist 10)																		
	Patient-centred/holistic care				✓	✓ *	✓	✓	✓				✓		✓				
	“It should be a multidisciplinary approach including the resident and their family in the discussion. I think that happens most of the time but sometimes it doesn’t. Yes, we can have guidelines and algorithms and everything, but it needs to be individualised, looking at all of their medicines and all of their health conditions.” (Pharmacist 2)																		
The Reasons for Reactive Deprescribing for PLWD	The PLWD not tolerating or coping with the practicalities of taking their medication				✓		✓	✓					✓ *	✓	✓	✓			
	“With dementia, people can have difficulties swallowing and things like that at the end of life and maybe we would have…to think about alternative routes of the medication.” (Community Pharmacist 2)																		
	Sharing of information with family members and/or PLWD	✓				✓	✓ *		✓				✓	✓	✓				
	“I generally don’t take away medicines without talking to families of patients with dementia. Most of them do not have the decision-making capacity on their own behalf. So we’re relying on the healthcare power of attorney.” (Provider, respondent 2)																		
	Side effects of medications	✓ *			✓	✓	✓	✓	✓	✓	✓		✓	✓	✓	✓			
	“I think people [should] be made aware there are problems. They may recognize themselves in this, ‘Oh, that’s why my mouth is so dry’ or, ‘I need to talk to the doctor about that.” (Non-paid carer, community ID # 129)																		
The Barriers and Facilitators for Proactive Deprescribing for PLWD	Prescribers following guidelines and evidence for deprescribing	✓			✓ *	✓		✓		✓			✓						
	“Well I think it would be useful if there was a guideline, particularly if we are talking about stopping medication. If there was a back-up, that there was evidence that [deprescribing] was the appropriate thing to do, then I think there would be a greater confidence in withdrawing medication at certain points in someone’s life.” (GP 5)																		
	Difficult if there are multiple specialists or providers of care for a PLWD	✓			✓			✓	✓		✓		✓		✓ *				
	“They continued to add on to them, not questioning the previous doctor or prescriber and going through, do we need all these things? So we’ve whittled it down to four or five things from probably 10 medications.” (Non-paid carer 10)																		
	Time constraints	✓	✓				✓	✓	✓				✓					✓ *	✓
	“I think the main thing we need, is time. If I think of the care home I look after, there’s 65 residents, so I’ve got to find… If I want to do it properly, I’d have find at least 65 hours a year to do it regularly, and follow-­ups. So it’s really quite hard to manage…So time is the biggest problem, and also, and time for care home staff. I mean, they’re as pressured as anyone else, for them to actually spend… Take an hour of their day out to talk about each one of their residents, again, is quite onerous on them.” (Care home 8, GP)																		
	Therapeutic or clinical inertia	✓			✓	✓	✓	✓ *			✓		✓		✓				
	“[Some clinicians] follow the path of least resistance…if nothing has changed and nothing is worse, do not stop anything. Just continue it because there’s no data against it, even though there may be no data for it.” (Cardiologist)																		
	Implications for the GP				✓	✓ *		✓	✓			✓	✓						
	The last thing a GP wants to do is change things around and then something bad happens, then it would be very easy for people to blame the GP for the decision on retrospect… if the patient is stable and there is no side effects, the tendency of many GPs is to just continue going. (GP 2, lipid-lowering agents)																		
	Withdrawal side effects of deprescribing or deterioration in condition	✓	✓		✓	✓	✓	✓	✓		✓	✓	✓	✓		✓ *		✓	
	“It became immediately clear that the medication had been giving her a quality of life that was valuable. Without it, her cognitive functioning and her ability to communicate was even worse, which was even more frustrating for her. We restarted it within a fortnight and she returned to her previous level.” (Rebecca)																		
	Lack of benefit to taking medication					✓	✓	✓	✓		✓		✓ *	✓			✓		
	“A statin is a long-term preventative measure, so if someone’s life expectancy is 6 months, a statin is not likely to do them a great deal of benefit.” (Hospital Geriatrician 12)																		
	Trust and distrust in patient/family - doctor relationship	✓			✓ *		✓		✓				✓	✓	✓	✓			
	“One lot [of doctors] will agree, one lot will disagree and I think sometimes you’ve got to be guided by the person you trust … if you trust your GP you can be guided by them.” (Non-paid carer 9)																		
	Fear of upsetting the doctor but the doctor wanting patient/family autonomy	✓ *			✓				✓										
	“My doctor should already know there’s some serious side effects or he should have already been warned there are serious side effects. So, if I take this to him, then what is he supposed to do with it?” (Non-paid carer, community ID # 133)																		
Difficulties of Deprescribing for the Family Members of PLWD	The burden	✓			✓						✓		✓		✓	✓	✓ *		
	“But it’s a real question isn’t it, as the dementia progresses, quality of life for both the person who has the dementia, and the carer, it’s a big issue. They live in a world of their own, it doesn’t worry them, it worries us.” (Focus group 4, non-paid carer, care home)																		
	Not wanting to “give up”	✓			✓	✓	✓	✓	✓ *				✓						
	“[The doctor] would say, ‘At your age… you probably have lived a good, long life.’… I didn’t like that because I would like to preserve her forever.” (Caregiver)																		
	Difficult and awkward conversations				✓				✓	✓ *									
	“It’s not an easy conversation to say, ‘I think your life expectancy is about 3 years and this statin is not going to benefit you.’” (Physician)																		
The Timing of Deprescribing Decisions for PLWD	Stages of dementia and quality of life and functional ability of the PLWD	✓			✓	✓	✓ *	✓	✓		✓	✓	✓	✓	✓	✓	✓		✓
	“When you’re 96, there might not be too many years down the road, so it’s probably important to see that somebody feels good. If you’re 35, yeah, you want to think about the long-term and consequences, so even though you think about the long-term consequences, I think it’s not as important for somebody who is very, very old.” (Caregiver, respondent 8)																		
	Dementia in palliative care and end-of-life care				✓ *	✓	✓	✓					✓						
	“People [with] end stage dementia would come in with pneumonia, come in with stuff that’s kind of getting towards more end of life care, but [it] was always really hazy … like do you keep giving them all the medications?” (Nurse 2)																		
	Wishes for the future				✓	✓							✓ *						
	“GPs are encouraged to do advance care planning with patients. If we have a prior discussion with the patient and/or their relatives that would be great and guide us as to how to make decisions… a lot of this comes down to communication with family, with carers, with the nursing homes and having it actually recorded.” (GP 3)																		

*Denotes which article the quote is from

Generally, the GP is the expert on diagnosing, treating, and preventing disease and the pharmacist is the expert on medication (in five studies). The nurse and/or the family member know the PLWD well, they are the expert of the PLWD as well as their advocate (discussed in twelve studies). The nurse has her nursing expertise to help support decision-making; however, family members may find their role challenging due to their lack of understanding of medicine and pharmacology:

*“With no medical training*,* I was made responsible for the gradual withdrawal or introduction of a large number of pretty heavy drugs*,* and I was really worried that I might make a mistake or miss some potentially dangerous side effect.”* (Joan; [43]).

In the home setting, it is usually a family member who will act as an advocate; they understand the PLWD’s normal behaviours and can “pick up” on signs when something is wrong, such as withdrawal side effects. However, if the PLWD lives in a care home, different family members will choose to be involved to varying degrees [41]. But family members do not always agree with one another, which can complicate deprescribing decision-making [40].

In a care home, the nurse and care workers will also be experts on the PLWD, which was stated in seven studies. This skill becomes more difficult and time-consuming as the severity of the dementia worsens [44, 49]. When communication becomes very difficult for the PLWD, and they are unable to express that they have possible side effects, there is a significant reliance on care workers and nurses to recognise issues [33]. Some unwanted side effects may never be recognised [41]. Although people may not experience side effects when they commence a medication, over time, side effects can start to occur [38].

It was evident throughout the review that although the GP had overall responsibility for deprescribing, it required a teamwork approach. Seven studies reported that the PLWD was central to this decision-making process and the care planned should be holistic.

#### The reasons for reactive deprescribing for PLWD

One of the main reasons quoted for deprescribing preventive medications was that the PLWD was no longer able to swallow their tablets, as the ability to swallow is often a problem as dementia progresses [40, 42, 43]. Alternative routes or liquid form may be considered, however, there is sometimes a reluctance to do this because the cost can be 50–100 times higher [40]. Two studies from the USA showed that for some families the cost of medication was a concern and for others it was not [36, 39].

Another reason stated for deprescribing medications is because the PLWD may not be able to manage their medication safely. If the PLWD lives on their own, certain drugs, such as anti-coagulants, may be considered too risky to prescribe, as they may take too many [37]. Some medications require monitoring, which may be considered too complicated for PLWD to incorporate into their lives [41, 42]. PLWD might refuse help with their medication due to a lack of comprehension of their condition or refuse to take their medications because they do not remember they are prescribed for them or understand their purpose [34, 40, 42, 43] .

The side effects of medications that PLWD were taking was another reason to deprescribe and was discussed in all the main eleven studies, by healthcare professionals, PLWD and their family carers. The sharing of information was discussed in seven of the studies, and the importance of the family understanding the side effects of medications, so they can recognise them if they are occurring [33, 36]. There were reports that stopping medications, often all preventive medication, caused improvement in PLWD’s wellbeing [43].

#### The barriers and facilitators for proactive deprescribing for PLWD

Overall, deprescribing for PLWD was considered complex:

*“I find these patients quite overwhelming…there’s lots of things going on and lots of different corners.”* (GP 12; [34]).

However, PLWD and their family members were more willing to deprescribe medications if they thought it might improve memory and reduce confusion [33, 36, 39].

Six studies in the review state guidelines and better training could improve deprescribing. They reported that there is not adequate guidance for older, multi-morbid patients or that guidelines did not fit each individual case due to their complexity. Then, there are situations where guidelines encourage prescribing. For example, GPs are financially incentivised in the UK under the Quality and Outcomes Framework to prescribe preventive medication such as statins and antihypertensives [40] and this may lead to a reluctance to deprescribe.

Seven studies discussed the added complexity if specialists had prescribed medications as the GP may not want to override the specialist’s decision [37, 40]. So, this may require liaising with specialists [40]. Conversely, hospital doctors feel that they have no control over what the GP chooses to do once a patient is discharged from hospital [40] and some do not want to interfere with the decisions of the GP [40, 42]. It was also reported that it is challenging to know when and why some medications were started and that this needs to be understood before medicines are deprescribed [41].

Deprescribing conversations are considered time-consuming due to their complexity, and this was stated in eight studies. Most GPs have a set time in which to see their patients:

*“I’ve got 15 minutes to talk about 10 things.”* (Physician; [38]).

In the allocated time for an appointment, deprescribing may not be a priority, causing therapeutic inertia [33].

Therapeutic inertia is a term used to describe not changing treatment when there is a need to. From a clinician’s perspective, therapeutic inertia happens because it is easier to do nothing, especially if there is nothing prompting a change [33, 34, 37]. Likewise, patients and family members also feel the same [33, 36, 39, 42]:

*“I would like to see her stay on all the medicines that have been prescribed for her until the day comes that she closes her eyes for the last time. I mean I would be afraid of what would happen if all her tablets were stopped.”* (Key Decision-Maker 12; [40]).

Family members often did not fully understand that medications may have side effects particularly as the PLWD gets older and frailer. They were more concerned that the PLWD would deteriorate if medications were stopped and therefore did not see the benefits of deprescribing (33–36, 38, 39, 47, 40, 42, 43]. There are some medications that may have side effects on withdrawal [44, 35–37, 43, 49], which make them also difficult to deprescribe, and some drugs should be tapered to reduce the risk of having withdrawal symptoms [43].

Although the decision to deprescribe medication should always be a shared decision (between healthcare professionals and the PLWD or family member), the onus falls on the GP predominantly if, later, that decision was considered to be incorrect. Six studies discussed this issue:

*“The doctor is too terrified to stop [medication] because if they have a heart attack or a stroke then they’re going to get blamed for it.”* (Pharmacist 8; [34]).

Although there were worries for themselves about being blamed by the family or worries about legal implications, there was also a fear of making the PLWD’s life worse [35, 47].

People are often told they will need to be on certain medications for life [37, 38]. By deprescribing these medications GPs are worried that the family will perceive them to be “giving up” on the PLWD or trying to shorten their life [33, 37, 38, 40]. But that sometimes means that GPs are influenced by the pressure from the family to continue medications the GP believes are unnecessary and will only deprescribe medication if that is what the family wishes [35, 38, 40].

The relationship with the GP has been discussed in eight of the articles and plays a significant role in how comfortable families feel about deprescribing. People, to varying degrees, do not understand their medications and rely on their doctors to make decisions for them:

*“I have full trust in my doctor*,* I don’t know anything about the medicines or how they work on the body.” (Key Decision-Maker 13;* [40]*)*.

However, this level of trust and confidence in their GPs’ expert knowledge may be truer for people from older generations [34] or where there is an established, long-term relationship [38].

Three studies expressed a reluctance to confront a doctor about deprescribing:

*“People are afraid to let their doctors know they have opinions of their medication and feeling like we’re telling a doctor what to do.”* (non-paid carer, 70; [33]).

However, doctors in the same study felt well-informed patients and families might enable deprescribing decisions.

#### Difficulties of deprescribing for family members of PLWD

Family members often feel isolated and burdened with caring for and making decisions for their loved ones with dementia, taking on more responsibility as the dementia progresses [48]. The process of deprescribing was mentioned as an additional burden in five of the studies, however three studies stated that reducing the burden of taking medication in their daily lives encouraged deprescribing [39, 40, 42].

Seven studies discuss how family members do not want to “give up” on the PLWD. They do not want to lose the person they love [33, 37]:

*“So the family*,* the poor family*,* the beneficiaries of the will are not going to say ‘please don’t give my vegetative state mother antibiotics for this chest infection or urine infection’ because they’ll feel guilty about it.”* (GP 10; [34]).

This makes deprescribing a difficult conversation to broach.

Although all studies implied deprescribing discussions were difficult, three explicitly focussed on the awkwardness of the conversation, causing a reluctance to start the discussion. Addressing issues such as death and futility of treatment are uncomfortable issues to raise with a PLWD, for both the family and the healthcare professional [34, 38]. To make these conversations easier, prescribers talked about providing reassurance that they would monitor the effects of deprescribing and restart medications if necessary [36, 38, 39].

#### The timing of deprescribing decisions for PLWD

Family members make decisions about their loved ones based on what they think the PLWD would do if they still had mental capacity [40]. However, it is difficult to know what the PLWD would have wanted, and then family members may use their own personal viewpoints to make judgements [50]. Likewise, healthcare professionals have their beliefs:

*“If I get seriously ill and suffer from dementia*,* please don’t fight for prolonging my life to eternity.”* (GP 2; [45]).

The GP would not want life-prolonging treatment continued to the end of life, but from this statement it is not clear exactly when to stop them.

The timing of deprescribing preventive medication is often based on functional abilities, such as:

- “*pretty impaired*” (GP3; [35]).
- “*highly disabled*” (hospital geriatrician 5; [40]).
- “*in a nursing home…not eating much…not communicative and…incontinent*” (primary care clinician; [37]).

But these are subjective terms. A guideline for deprescribing certain medications at different stages of dementia may be useful [34], but that would need safeguards to ensure the correct stage of dementia was confirmed [35].

When someone is suitable for palliative care, this may trigger a medication review where their medications are changed to those controlling symptoms and pain management rather than prolonging life [40]. However, PLWD do not receive the same level of palliative care as cancer patients [34]. The considered need for palliative care in dementia varies dramatically from one doctor to another. Some think that when diagnosed with dementia the principles of palliative care should be started. In contrast, other doctors think that it is too soon but recognise that specifying when palliative care should start is difficult due to dementia having an unpredictable trajectory [35–37, 40]. The correct time to deprescribe preventive medication for long-term benefit, such as statins, is therefore difficult to predict.

Even at the end of life, decision-making for deprescribing medications is unclear:

*“People [with] end-stage dementia would come in with pneumonia…but [it] was always really hazy…like*,* do you keep giving them all the medications?”* (Nurse 2; [34]).

Some doctors even think that at the end of life, preventive medications should continue, as a heart attack or stroke would be an additional burden on the PLWD [40].

Three studies report that knowing what the PLWD wished for using end-of-life care plans or advance care planning makes care choices easier when the PLWD no longer has mental capacity [34, 35, 40]. This was stated by family members, GPs, nurses and pharmacists and was considered to take the pressure off the prescriber, as they were able to base their decisions on what the PLWD had requested [34].

## Discussion

This systematic review has identified the following main themes: the decision-makers of deprescribing, reactive reasons to deprescribe, barriers and facilitators to proactive deprescribing, difficulties for family members and the timing of deprescribing decisions. Deprescribing preventive medication for PLWD was considered difficult for both healthcare professionals and family members.

**Fig. 2:**
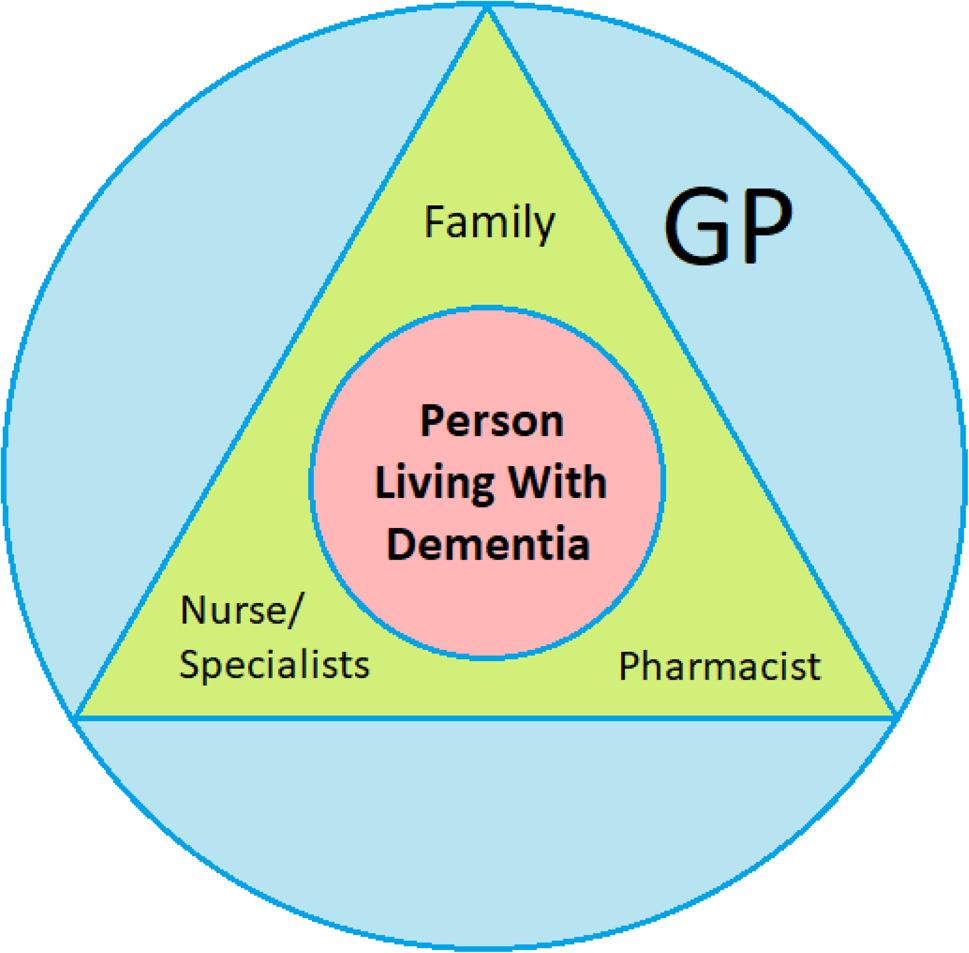
The stakeholders involved in deprescribing decision-making for PLWD who lack mental capacity for these decisions: The PLWD as the central focus, the GP with overarching responsibility, supported by family, nurse (or care workers in residential homes)/specialists and pharmacist

GPs are the main responsible person for making deprescribing decisions, and this has been found in similar systematic reviews [17–19]. GPs may be supported by nurses, specialists, pharmacists, and family members to try to make choices that the PLWD would have wanted (Fig. 2). In these similar systematic reviews, one of the barriers to deprescribing, not specific to PLWD, was poor relationships amongst the multidisciplinary team. However, our systematic review found only one example of this. The roles seem to be clearer when someone no longer has the mental capacity to make deprescribing decisions and a recent realist review showed that deprescribing is more effective if there are clearly defined roles for the decision-makers [51].

A lack of guidance was one of the main factors recognised in our review that made deprescribing difficult. Another systematic review [52] also highlighted the same problems: that guidelines are disease-specific and lack evidence for older people, multi-morbidity and drug interactions. Other reviews discuss how medication guidelines should incorporate how to deprescribe not just how to prescribe [53, 54]. However, our review showed that although guidance and evidence could be improved to help support deprescribing decision-making, it is unlikely to cover every individual’s complex circumstances:

*“The guidance is important but it’s only one part of what should be a complex management approach.” (old age psychiatrist 1;* [35]*)*.

In addition, GPs must consider the General Medical Council [55], which states they should always continue to prolong life unless the patient requests otherwise, complying with the Human Rights Act 1998, Article 2 [56] – the right to life. However, the World Health Organization [57] classes dementia as an illness that requires a palliative approach which should not “hasten nor postpone death” and this implies GPs should not be aiming to prolong life for PLWD.

The STOPP/START (Screening Tool of Older Persons’ Prescriptions and Screening Tool to Alert to Right Treatment) criteria [58] provides guidance for deprescribing potentially inappropriate medicines to people over the age of 65. A similar idea could be developed for PLWD, starting with the early stages of dementia (which would be like the STOPP/START criteria) and then to the middle and later stages as PLWD become frailer. The latter stages may match the STOPPFrail guidance [59]. It would be important to ensure that all healthcare professionals involved in deprescribing are able to use the tool [60], as deprescribing requires a team approach [61].

One of the barriers identified was that when doctors start medications, the patient is told that it will need to be taken for life. This was reported in other systematic reviews [17–19]. When another doctor then talks about deprescribing this medication later in life, the patient (or family) feels that this contradictory advice is unsound. One narrative review suggests a change in prescribing culture where every medication prescribed has a “time-to-review” [14]. Our systematic review recognised other useful information would be “reason-for-starting” and “date-started”, as past medical history is not always easy to ascertain, and it was not clear whether it was being given for symptoms or to prolong life [62]. Prescriptions could have this information recorded on them, not only for other and future healthcare professionals but also for the patient and their families [63].

Other barriers found in our systematic review showed that deprescribing was seen as being given less care. This is mirrored in another systematic review, where the attitude to medication was “more is better” and “a pill for every ill” [52]. “Polypharmacy” could be re-framed as an illness, so that everyone would try to prevent polypharmacy as much as they would try to prevent a stroke [64].

When someone cannot explain or express that they have side effects of preventive medications, it could be questioned whether a palliative-only approach is more appropriate. Conversely, a narrative review [16] found that GPs were reluctant to deprescribe medications when someone was unable to communicate the withdrawal effects. Future research could explore the concept of deprescribing PLWD’s preventive medication before they are unable to communicate.

One of the main issues throughout the studies was determining when a PLWD was considered to be palliative; when only palliative, symptom-controlling treatments would be given and preventive medication would be deprescribed [18]. However, before someone loses capacity, they might be able to define when this moment would be for them, which could be recorded in advance care planning (although some people may wish to continue preventive medication until death). Our systematic review shows that stakeholders struggle to make deprescribing decisions, and it would have been helpful to know what the PLWD would have wanted. It has been suggested that the burden of making decisions for family members is lessened with the use of advance care planning [65]. In care home settings, the use of advance care planning and ReSPECT (Recommended Summary Plan for Emergency Care and Treatment [66]) forms, has increased over the past few years [67]. So advance care plans are already used to state whether someone wants to be resuscitated, in what situations they would want to go to hospital (including intensive care) and in what situations they would rather die at home. Advance care plans incorporate decisions around the use of antibiotics, but, as yet, does not include deprescribing preventive medication [68]. Further research could explore this topic.

### Strengths and limitations

The original database search found 17 out of the 18 included studies. So, in the further searches (including citation mining and reference checks) of over 1400 studies, only one other study was found ([40]). This shows that the search strategy was robust.

Only English-language articles were included in our systematic review. But, given most deprescribing research has been conducted in Australasia, Europe and North America, then published in English-language journals, this is not considered a significant limitation. However, as all the studies were conducted in Westernised countries, our review’s findings may not be transferable to non-Westernised countries. These countries may have different approaches to medication management, from both the different healthcare systems as well as from cultural perspectives and beliefs affecting their attitudes to deprescribing [69]. Also, the review presents dementia as one homogeneous disease, as this was how it was presented in the 18 studies. So, the review’s findings may not be applicable to every type of dementia.

Robust screening methods were used with a second reviewer and support from an experienced medical librarian. The second reviewer independently checked at least 20% of the quality assessments and coding; with no major differences found. The review team, with content expertise, was involved throughout the systematic review process to ensure the review was on schedule, the correct processes were followed, and the findings were valid.

## Conclusion

GPs, nurses and pharmacists consider deprescribing preventive medication in dementia a time-consuming and complicated issue. Guidance is often unclear for this complex group and deprescribing conversations are difficult. Deprescribing is made even harder due to an unclear prognosis and trajectory of dementia. However, our review suggests the roles professionals and family members tend to adopt in decision-making for PLWD are clearer and this may facilitate deprescribing.

Shared decision-making may take some of the responsibility from the prescriber, usually the GP, but the prescriber still has overall accountability for deprescribing. Advance care planning for deprescribing, would further reduce the burden on families and healthcare professionals, by supporting shared decision-making involving the views of the PLWD.

## Supplementary Information

Supplementary Material 1.

Supplementary Material 2.

## Data Availability

Appendix 1 contains the ENTREQ statement, the search strategy for Embase and the inclusion and exclusion criteria. Search strategies for all the databases can be found with the published protocol (23). Further information is available from the corresponding author on reasonable request.

## Abbreviations

CASP: Critical Appraisal Skills Programme
ENTREQ: Enhancing transparency in reporting the synthesis of qualitative research
GP: General practitioner
PLWD: People/person living with dementia
PRISMA: Preferred Reporting Items for Systematic reviews and Meta-Analyses
ReSPECT: Recommended Summary Plan for Emergency Care and Treatment
STOPP/START: Screening Tool of Older Persons’ Prescriptions and Screening Tool to Alert to Right Treatment
UK: United Kingdom
USA: United States of America
